# Can a toilet promote virus transmission? From a fluid dynamics perspective

**DOI:** 10.1063/5.0013318

**Published:** 2020-06-01

**Authors:** Yun-yun Li, Ji-Xiang Wang, Xi Chen

**Affiliations:** 1 Key Laboratory of Energy Thermal Conversion and Control of Ministry of Education, School of Energy and Environment, Southeast University, Nanjing 210096, People’s Republic of China; 2 Jiangsu Key Laboratory of Micro and Nano Heat Fluid Flow Technology and Energy Application, School of Environmental Science and Engineering, Suzhou University of Science and Technology, Suzhou 215009, People’s Republic of China; 3 College of Electrical, Energy and Power Engineering, Yangzhou University, Yangzhou 225009, People’s Republic of China

## Abstract

Currently, a novel coronavirus named “SARS-CoV-2” is spreading rapidly across the world, causing a public health crisis, economic losses, and panic. Fecal–oral transmission is a common transmission route for many viruses, including SARS-CoV-2. Blocking the path of fecal–oral transmission, which occurs commonly in toilet usage, is of fundamental importance in suppressing the spread of viruses. However, to date, efforts at improving sanitary safety in toilet use have been insufficient. It is clear from daily experience that flushing a toilet generates strong turbulence within the bowl. Will this flushing-induced turbulent flow expel aerosol particles containing viruses out of the bowl? This paper adopts computational fluid dynamics to explore and visualize the characteristics of fluid flow during toilet flushing and the influence of flushing on the spread of virus aerosol particles. The volume-of-fluid (VOF) model is used to simulate two common flushing processes (single-inlet flushing and annular flushing), and the VOF–discrete phase model (DPM) method is used to model the trajectories of aerosol particles during flushing. The simulation results are alarming in that massive upward transport of virus particles is observed, with 40%–60% of particles reaching above the toilet seat, leading to large-scale virus spread. Suggestions concerning safer toilet use and recommendations for a better toilet design are also provided.

## I. INTRODUCTION

Since December 2019, a large outbreak of atypical pneumonia caused by a novel coronavirus named “SARS-CoV-2” occurred in Wuhan, Hubei Province of China, resulting in millions of people infected within a short period of time across the world.^1^ Since then, SARS-CoV-2 has spread amazingly rapidly, with more than 4.9 × 10^6^ confirmed cases worldwide before 20 May 2020, posing a huge public health challenge.^2^ Because of the serious economic losses and panic that it has caused, SARS-CoV-2 was declared a worldwide pandemic by the World Health Organization (WHO) on 11 March 2020.^3^ The main transmission routes of SARS-CoV-2 are droplets and direct contact, but some patients have developed gastrointestinal symptoms such as diarrhea and vomiting, showing that the virus can survive in the digestive tract.^4^ It is worth mentioning that, in March 2020, a research team from Sun Yat-Sen University found that fecal samples from some confirmed patients tested positive by nucleic acid detection, which provides evidence that SARS-CoV-2 has the possibility of fecal–oral transmission.^5^

Numerous past studies have demonstrated that human coronaviruses (which are considered a major global public health threat), such as the severe acute respiratory syndrome-related coronavirus (SARS-CoV) and the Middle East respiratory syndrome-related coronavirus (MERS-CoV), are characterized by fecal–oral transmission.^6,7^ In addition, as common intestinal pathogens, norovirus and rotavirus can spread easily through the fecal–oral route because their main symptoms are acute diarrhea and vomiting.^8^ It can be concluded that fecal–oral transmission is not a unique feature of the currently raging SARS-CoV-2 but a common transmission channel for most viruses. Therefore, blocking the path of fecal–oral transmission can reduce the probability of cross-infection in surrounding areas, thus suppressing the global spread of emerging and re-emerging viruses.

According to the characteristics of fecal–oral transmission, there will be a large amount of viruses within a toilet when a confirmed case uses it. Thus, toilets should be regarded as one of the infection sources. However, toilet design and use are often neglected. Improper toilet use will cause cross-infection through fecal–oral transmission among people if precautionary measures are not taken. Such cross-infection usually occurs in family bathrooms and public washrooms. A confirmed case usually remains at home for isolation, where shared use of a bathroom is inevitable. The daily flow of people in a public washroom is stunningly large: thus, a confirmed case may cause a massive number of infections. For these reasons, investigation of toilets in the context of epidemic prevention is imperative.

Toilets have been investigated extensively, since they are among the daily but dangerous necessities of life, with important effects on human health and wellbeing. By installing a variety of sensors in a toilet, Park *et al.*^12^ designed a smart toilet that can be used for screening, diagnosis, and longitudinal monitoring of specific groups of patients by conducting long-term analyses of their excreta. Flores *et al.*^9^ analyzed the biogeographical distribution of bacteria across public washroom surfaces, where up to 19 bacterial phyla were found. In particular, gut-associated taxa were detected on toilet surfaces. They also adopted the SourceTracker algorithm and identified the human skin as the primary source of the detected bacteria. To investigate the sanitary habits of Chinese people in public lavatories in Hong Kong, Wu *et al.*^10^ conducted qualitative interviews and a self-administered questionnaire survey among local residents. Qualitative data analysis revealed that the public had a number of poor hygiene habits when using the toilet, including spitting into it and flushing without covering the lid, which can increase the risk of bacterial and viral infections. Hennigs *et al.*^11^ tested a prototype of a non-fluid-based mechanical toilet flush by means of a field test. They concluded that a waterless flushing has the potential to reduce the risk of pathogen transmission because it can create a physical barrier between feces and the user by sealing the feces. From the literature review above, it can be shown that current toilet-oriented studies have an emphasis on infectious disease tracking, design optimization, and public health management. It can also be noted that a new design of waterless toilets could suppress the transmission of pathogens. However, considering the popularity and widespread use of normal water-based toilets, large-scale introduction of waterless toilets would take some time. At present, therefore, with the aim of raising public awareness of the fact that bad habits when using water-based toilets can increase the risk of virus transmission, it is important to understand the mechanisms involved. Unfortunately, to date, there have been very few mechanistic studies.

As can be seen from our daily experience, flushing a toilet can cause violent turbulence, which will aid large-scale spread of viruses present in the toilet bowl. It has also been shown that flushing the toilet without putting the lid down is a bad habit.^10^ Health risks brought by the improper toilet flushing were also identified by Hamilton *et al.*^13^ and Aithinne *et al.*,^14^ where aerosol particles carrying viruses could spread indoors. However, a clear and comprehensive explanation has not been given to the public, and therefore, there has not been widespread acceptance and implementation of this simple precaution. This paper adopts the method of control-volume-based computational fluid dynamics (CFD) to explore the fluid flow characteristics during toilet flushing and demonstrates how flushing promotes the spread of viruses. Given that flushing involves a strong interaction between the air and the liquid, the volume-of-fluid (VOF) model, which has been successfully applied for the simulation of various multiphase flows such as droplet spreading on surfaces,^15^ gas–liquid jet processes,^16^ and droplet formation and detachment,^17^ is used to simulate the flushing processes of two different types of siphon toilets that are in widespread use: a single-inlet model and an annular one. During the flushing process, water enters the bowl from a tank under the action of pressure and mixes with the water seal. This generates turbulent motion, which drives dramatic changes in airflow. In the simulations presented here, the movements of virus particles along with the two-phase flow are investigated using a coupled method based on the VOF model and discrete phase model (DPM). Finally, suggestions to reduce the risk of infection through fecal–oral transmission during the use of the toilet will be provided. The results presented in this paper should not only deepen our knowledge about gravity-driven two-phase flow dynamics but also provide a basis for promoting public awareness of how to use toilets in a more sensible way and thus teach the public to do their part to prevent epidemic transmission. Moreover, it will shed light on the novel design of a better toilet.

The remainder of this paper is organized as follows. The two different types of toilet models are described in detail in Sec. II. In Sec. III, the mathematical formulations of the models utilized in this paper are provided. Section IV presents the relationships involved in the CFD simulations, including meshing, boundary conditions, and simulation cases. The simulation results are presented in Sec. V, together with a discussion. The main conclusions, including suggestions for more appropriate toilet use and better toilet design, are given in Sec. VI.

## II. TOILET MODEL STRUCTURES

**FIG. 1.**
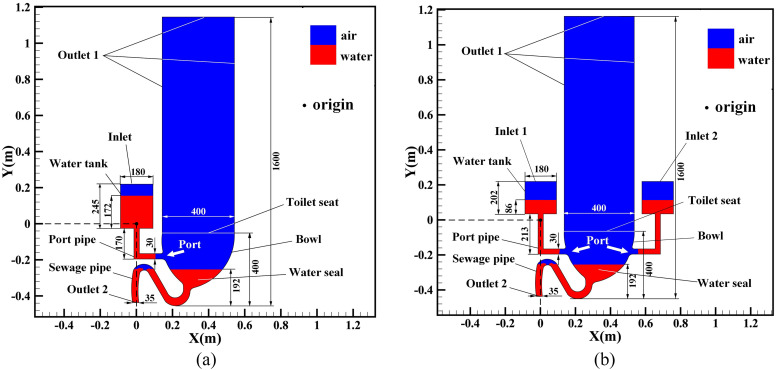
Structures and overall dimensions of siphon toilets: (a) single-port toilet; (b) double-port toilet (units: mm).

The focus of this paper is the flushing process of common siphon toilets, as shown in Fig. 1, which schematically depicts two simplified two-dimensional siphon toilets, of single-port and double-port types, respectively. The single-port toilet can be used to simulate single-inlet flushing and the double-port toilet annular flushing. In Fig. 1, the red areas represent the liquid phase and the blue areas the air phase. Only the red and blue areas excluding the white areas in Figs. 1(a) and 1(b) are the calculation regions of these two models.

As can be seen in Fig. 1(a), the single-port toilet uses only one tank to fill the bowl with water, with the flushing port on one side of the bowl, and the width × height of the water tank is 180 × 245 mm^2^. The height and width of the toilet seat are both 400 mm. The diameters of the port pipe and the sewage pipe are 30 mm and 35 mm, respectively, and the length of the vertical port pipe directly connected to the water tank is 170 mm. To observe how the flow dynamics inside the bowl affects the airflow over the toilet and the movement of virus particles out of the toilet during the post-flushing stage, the air zone above the toilet was studied, with the distance between the top of the air zone and the ground being 1600 mm. Another reason of setting 1600 mm is that the average height of mouths of common people is usually at 1600 mm, which is critical to the fecal–oral transmission. The initial height of the water level in the tank is 172 mm, and a water seal is located at the bottom of the basin at a height of 192 mm, while the remaining area is filled with air. The single-port toilet has one inlet and two outlets (outlets 1 and 2). The inlet is located at the top of the water tank, outlet 1 includes the top and both sides of the air zone, and outlet 2 is the bottom end of the sewage pipe, where the sewage is discharged.

To simulate the widely used annular flushing process, a toilet with two identical tanks to feed water into the bowl at the same time is considered, as depicted schematically in Fig. 1(b), where the two inlets are symmetrically distributed on each side of the bowl. To ensure that the total amount of water and its gravitational potential energy in the two tanks are identical to those in the single-port toilet at the beginning of the flushing process, the amount of water in each tank is one-half that of the single-port toilet and the tanks are raised by 43 mm, which is one-quarter of the height of the water in the tank of the single-port toilet. Therefore, the width × height of each water tank is 180 × 202 mm^2^, with the water level at a height of 86 mm. The lengths of the two vertical parts of the port pipes connecting the tanks to the toilet both become 213 mm. The other dimensions are the same as those of the single-port toilet. The double-port toilet has two inlets (inlets 1 and 2), which are, respectively, on the top of the two tanks, and two outlets (outlets 1 and 2), which are identical to those of the single-port toilet.

## III. MATHEMATICAL FORMULATION

The toilet flushing process involves continuous deformation of the gas–liquid interface, in a process of free surface fluid flow. Therefore, the VOF model, which is able to deal with the dynamics of free boundaries,^18^ is adopted to track the two-phase interface in this paper. In addition, a turbulence model, the realizable *k*-*ε* model,^19^ is used to simulate the flow patterns of the fluid, and the movement of virus particles under the action of flushing is tracked by the DPM, which is a Lagrangian tracking approach.^20^

### A. VOF model

The volume fractions of all phases are summed up in each control volume in the VOF formulation. Depending on the values of the volume fraction, the variables and properties in any given grid represent either a single-phase or a two-phase state. For the specific simulation of toilet flushing in this paper, the volume fraction of the water phase, which is denoted *α*_*w*_, has three possible conditions, as shown in Table I.

**TABLE I.** Three possible conditions on *α*_*w*_.

Condition 1	*α*_*w*_ = 0	The cell is empty of water
Condition 2	*α*_*w*_ = 1	The cell is full of water
Condition 3	0 < *α*_*w*_ < 1	The cell contains the interface between water and air

By solving the continuity equation for the volume fraction of the phases, the interface between the phases can be tracked. Based on geometrical advection using piecewise linear interface calculation (PLIC),^21^ the continuity equations (N-S equations) for the water and air phases are

$$\frac{\partial \alpha_{w}}{\partial t}+{\vec{v}}_{w}\cdot \nabla \alpha_{w}=\frac{S_{\alpha_{w}}}{\rho_{w}},$$ (1)

$$\frac{\partial \alpha_{a}}{\partial t}+{\vec{v}}_{a}\cdot \nabla \alpha_{a}=\frac{S_{\alpha_{a}}}{\rho_{a}},$$ (2)

where *t* is the time, *α*_*w*_ and *α*_*a*_ are the volume fractions of water and air, ${\vec{v}}_{w}$ and ${\vec{v}}_{a}$ are the velocity vectors of water and air, and *ρ*_*w*_ and *ρ*_*a*_ are the densities of water and air. Under the assumption that the air does not dissolve in water, the source terms $S_{\alpha_{w}}$ and $S_{\alpha_{a}}$ are zero. In addition, *α*_*w*_ and *α*_*a*_ are constrained by the following equation:

$$\alpha_{w}+\alpha_{a}=1.$$ (3)

In the transport equations, the properties of each control volume are determined by the volume fraction of the component phases. For condition 3 in Table I, the mixture density *ρ* and viscosity *μ* in each cell are calculated as follows:^22^

$$\rho =\alpha_{a}\mu_{a}+\left(1-\alpha_{a}\right)\rho_{w},$$ (4)

$$\mu =\alpha_{a}\mu_{a}+\left(1-\alpha_{a}\right)\mu_{w}.$$ (5)

In the VOF frame, the momentum equation is solved in the whole calculation region, where the obtained velocity field is shared among the phases. Depending on the mixed variables such as *ρ* and *μ*, the momentum equation, which is two-way coupled with Eqs. (1) and (2), is

$$\begin{array}{l} \frac{\partial }{\partial }\left(\rho \vec{v}\right)+\nabla \cdot \left(\rho \vec{v}\vec{v}\right)=-\nabla p+\nabla \cdot \left[\mu \left(\nabla \vec{v}+\nabla {\vec{v}}^{T}\right)\right]+\rho \vec{g}+F_{sf}, \end{array}$$ (6)

where *p* is the static pressure, $\vec{v}$ is the velocity vector of the mixed fluid, $\rho \vec{g}$ is the gravitational body force, and *F*_*sf*_ represents the source terms of surface tension forces.

To solve the continuity equations and the momentum equation, the continuum surface force (CSF) model, which transforms the surface tension forces to a volumetric force, is used to calculate the surface tension force via the following expression:^15^

$$F_{sf}=2\sigma \frac{\bar{\rho }\cdot \nabla \alpha_{w}}{r\left(\rho_{a}+\rho_{w}\right)},$$ (7)

where σ is the surface tension coefficient (0.0725 N/m), $\bar{\rho }$ is the volume-averaged density, which is calculated from Eq. (4), and *r* is the liquid–gas interface radius, which is calculated from

$$\frac{1}{r}=\nabla \cdot \frac{\nabla \alpha_{w}}{\left|\nabla \alpha_{w}\right|}.$$ (8)

### B. Turbulence model

It is acknowledged that toilet flushing is a strongly turbulent process. Therefore, an appropriate turbulence model is required for its description. The realizable *k*-*ε* model, which can provide acceptable accuracy at a relatively low computational cost,^23^ is adopted in this paper. The governing equations to be solved for the turbulent kinetic energy *k* and dissipation rate *ε* are

$$\begin{array}{ccc} \frac{\partial \left(\rho \varepsilon \right)}{\partial t}+\frac{\partial \left(\rho \varepsilon u_{i}\right)}{\partial x_{i}} & = & \frac{\partial }{\partial x_{i}}\left[\left(\mu +\frac{\mu_{t}}{\sigma_{\varepsilon }}\right)\frac{\partial \varepsilon }{\partial x_{i}}\right]+\rho c_{1}S_{\varepsilon }\\ & & -\rho c_{2}\frac{\varepsilon^{2}}{k+\sqrt{v\varepsilon }}+c_{1\varepsilon }\frac{\varepsilon }{k}c_{3\varepsilon }G_{b}, \end{array}$$ (9)

$$\begin{array}{ccc} \frac{\partial \left(\rho \varepsilon \right)}{\partial t}+\frac{\partial \left(\rho \varepsilon u_{i}\right)}{\partial x_{i}} & = & \frac{\partial }{\partial x_{i}}\left[\left(\mu +\frac{\mu_{t}}{\sigma_{\varepsilon }}\right)\frac{\partial \varepsilon }{\partial x_{i}}\right]+\rho c_{1}S_{\varepsilon }\\ & & -\rho c_{2}\frac{\varepsilon^{2}}{k+\sqrt{v\varepsilon }}+c_{1\varepsilon }\frac{\varepsilon }{k}c_{3\varepsilon }G_{b}. \end{array}$$ (10)

Formulas for the variables in Eqs. (9) and (10) are presented in Table II, where $V_{⊥g}$ is the velocity perpendicular to the gravity.

**TABLE II.** Values and physical meanings of the variables in Eqs. (9) and (10).

Variable	Calculation formula	Physical meaning
*μ*_*t*_	$\rho c_{\mu }\frac{k^{2}}{\varepsilon }$	Turbulent viscosity
*M*_*t*_	$\sqrt{k/a^{2}}$	Turbulent Mach number
*G*_*k*_	$\mu_{t}\left(\frac{\partial v_{i}}{\partial x_{j}}+\frac{\partial v_{j}}{\partial x_{i}}\right)\frac{\partial v_{i}}{\partial x_{j}}$	Turbulent kinetic energy produced by the average velocity gradient
*G*_*b*_	$\beta g\frac{\mu_{t}}{\text{P}{\text{r}}_{t}}\frac{\partial T}{\partial y}$	Turbulent kinetic energy produced by the buoyancy force
*Y*_*M*_	$2\rho \varepsilon M_{t}^{2}$	Fluctuation effect on the total dissipation rate in a compressible flow
*σ*_*k*_	1	Model constant
*σ*_*ε*_	1.2	Model constant
*c*_1_	$\text{max}\left(0.43,\;\frac{\eta }{\eta +5}\right)$	Model constant
*η*	$\frac{k}{\varepsilon }\sqrt{\left(2S_{ij}\cdot S_{ij}\right)}$	Turbulent time scale divided by the time-averaged strain rate of air
*S*_*ij*_	$\frac{1}{2}\left(\frac{\partial u_{i}}{\partial x_{j}}+\frac{\partial u_{j}}{\partial x_{i}}\right)$	Time-averaged strain rate of air
*c*_2_	1.9	Model constant
*c*_1*ε*_	1.44	Model constant
*c*_3*ε*_	$\text{tanh}\left(V_{⊥g}/v\right)$	Buoyancy effect on *ε*

### C. Discrete phase model

Frequently used in particle tracking, the DPM has been successfully applied in various areas, such as a droplet movement in spray cooling,^24^ where numerous fine droplets are ejected through a tiny orifice,^25–27^ and a dust particle diffusion movement, which can be used to reinforce pollution control.^28^ Recently, Dbouk and Drikakis^29^ adopted the DPM to simulate a human-cough-induced droplet movement, which shares similarities with the investigation in this paper. The DPM is adopted in this paper to stimulate the virus particle movement under the effect of toilet flushing. The flow pattern of a particle is calculated from the following equation:

$$\frac{d{\vec{v}}_{d}}{dt}=F_{D}\left({\vec{v}}_{x}-{\vec{v}}_{d}\right)+\frac{\vec{g}\left(\rho_{d}-\rho \right)}{\rho_{d}}+{\vec{F}}_{y}\left(x=w\;or\;a\right).$$ (11)

The diameter *d* of the particles carrying viruses (henceforth “virus particles”) is between 1 *μ*m and 10 *µ*m^30^ (the viruses themselves are much smaller, of the order of 100 nm), and so the Stokes drag force equation is adopted to calculate *F*_*D*_ as follows:

$$F_{D}=\frac{18\mu }{d^{2}\rho_{d}C_{c}},$$ (12)

where the Cunningham coefficient *C*_*c*_ under atmospheric conditions is calculated to be 1 from the following equation:^31^

$$C_{c}=1+\frac{2\lambda }{d}\left(1.257+0.4e^{-\left(1.1d/2\lambda \right)}\right),$$ (13)

where *λ* is the gas means free path.

In addition, because of the size of the bioaerosol particles, the Brownian force and Saffman lift force are taken into account in the form of ${\vec{F}}_{y}$, for which a detailed formula can be found in Li and Ahmadi’s work.^33^

In addition, the discrete random walk model that is clearly described in Jin’s work^32^ is adopted to calculate the turbulent diffusion of the aerosol particles where the actual transient velocities of the water and air phases in Eq. (11) can be calculated by the following equation:

$${\vec{v}}_{x}={\bar{\vec{v}}}_{x}+{{\vec{v}}_{x}}^{\prime }\left(x=w\;or\;a\right),$$ (14)

where ${\bar{\vec{v}}}_{x}$ is the time-averaged velocity of the fluid and ${{\vec{v}}_{x}}^{\prime }$ is the pulsating velocity due to the turbulence.

## IV. COMPUTATIONAL MODEL

### A. Assumptions

In order to simulate the particle movement during toilet flushing, several assumptions are adopted in this manuscript: (1) There is no heat and mass transfer between the aerosol particles and the air. (2) The generation of the aerosol particles is ignored. (3) The size and other physical properties of the aerosol particles remain constant during simulation. (4) The temperature is assumed to be 20 °C. (5) Effects of feces in the toilet are ignored.

### B. Mesh condition

**FIG. 2.**
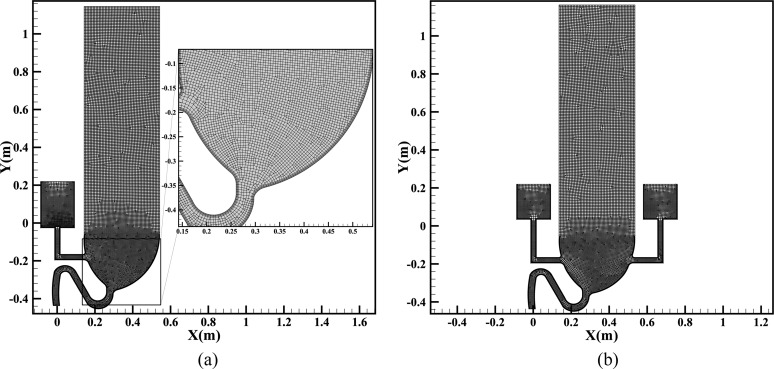
(a) A close view of the mesh inside the single-port toilet model; (b) mesh condition for the double-port toilet model.

Figure 2 presents the mesh conditions of the two computational models, where an encryption method near the wall and a global encryption within the bowl are adopted. Since the overall structures of the two models are similar, an identical mesh generation method is used, in which the total mesh number of the single-port toilet model is 17 708 and that of the double-port model is 19 135. The network quality of the meshes is above 0.9 throughout, representing a perfect mesh quality. The results of a mesh sensitivity analysis for the double-port model are presented in Table III, from which it can be seen that a mesh number of 19 135 can provide both accuracy and computational economy.

**TABLE III.** Mesh sensitivity analysis of the double-port model with a center point whose coordinate is (0.46, −0.21).

Mesh number	8034	17 708	21 834	27 623
Velocity magnitude (m/s)	9.456	9.629	9.623	9.627
Gauge pressure (Pa)	−41.670	−43.098	−43.064	−43.073

### C. Simulation conditions, cases, solution, and process

**FIG. 3.**
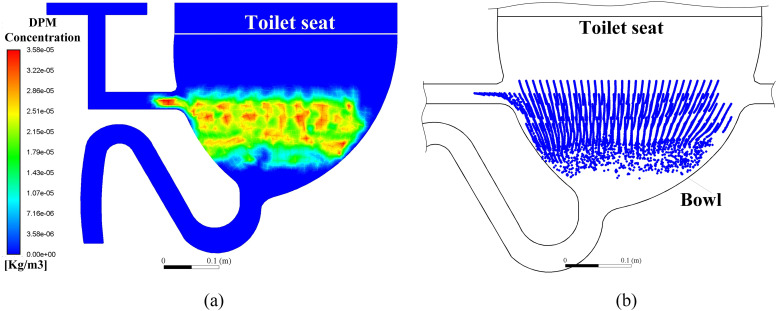
Initial conditions of the aerosol particle distributions of the two toilet models shown (a) in the form of particle concentration for the single-port toilet and (b) as discrete particles for the double-port toilet.

Two cases are simulated for each toilet model: (1) transient state of toilet flushing and (2) transient state of aerosol particles moving under the influence of flushing. Detailed information on the CFD simulation and the boundary conditions is given in Table IV, and the initial conditions of the aerosol particle distributions of the two toilet models are presented in Fig. 3. Figure 3(a) shows the particle concentration in the single-port toilet, while Fig. 3(b) shows the discrete particle distribution in the double-port toilet. Note that the initial particle distributions in the two toilets are comparable, with the total number of particles being 6000 for each model. The critical physical parameters of the aerosol virus particles are obtained from Gupta *et al.*^34^ and Wong *et al.*^35^

**TABLE IV.** CFD simulations and boundary conditions.

Model type	Single-port toilet	Double-port toilet
Mesh number	17 708	19 135
Materials	Water–liquid (primary phase), air (secondary phase)	
Operating conditions	Pressure: 101 325 Pa, air density: 1.225 kg/m^3^	
	Pressure-inlet *α*_*a*_ = 1, gauge total pressure 0 Pa. Turbulence specification method:	
Inlet	intensity and hydraulic diameter, with turbulent intensity 5% and hydraulic diameter 0.18 m.	
	Inlets 1 and 2 have the same boundary conditions for both toilets	
	Pressure-outlet *α*_*a*_ = 1. Backflow pressure specification:	
Outlet 1	total pressure. Turbulence specification method: intensity and hydraulic diameter,	
	with turbulent intensity 5% and hydraulic diameter 0.4 m. Escape boundary type	
	Pressure-outlet *α*_*a*_ = 1. Backflow pressure specification:	
Outlet 2	total pressure. Turbulence specification method: intensity and hydraulic diameter,	
	with turbulent intensity 5% and hydraulic diameter 0.035 m. Escape boundary type	
Bowl	No-slip wall boundary, reflect boundary type	
Aerosol particle^14,34,35^	Particle diameter 8.6 *µ*m, total number 6000, particle density 1100 kg/m^3^	

To conduct a comparative study between single-inlet flushing and annular flushing, the following simulation procedures are used for both toilet models: (1) AutoCAD software is used to construct geometric models; (2) CFD-ICEM software is used to set the boundary types and to generate the mesh; (3) Ansys-Fluent 19.2 software is used to simulate toilet flushing and the resulting particle movement; and (4) data extraction and analysis are carried out, and the simulation results for the two models are compared.

To solve the coupling between pressure and velocity, the pressure-implicit with splitting of operators (PISO) solution is adopted, which is highly recommended for all transient flow calculations. Spatial discretization of the gradient is selected to be least-squares cell-based, pressure is PRESTO, momentum is second-order upwind, volume fraction is Geo-Reconstruct, and other parameters such as turbulent kinetic energy and turbulent dissipation rate are power-law. The time step size is 0.0005 s, which proves to have rapid convergence.

## V. RESULTS AND DISCUSSION

Analyses of the toilet flushing process and the associated particle movement for each toilet model are presented in this section.

### A. Analysis of flushing process

**FIG. 4.**
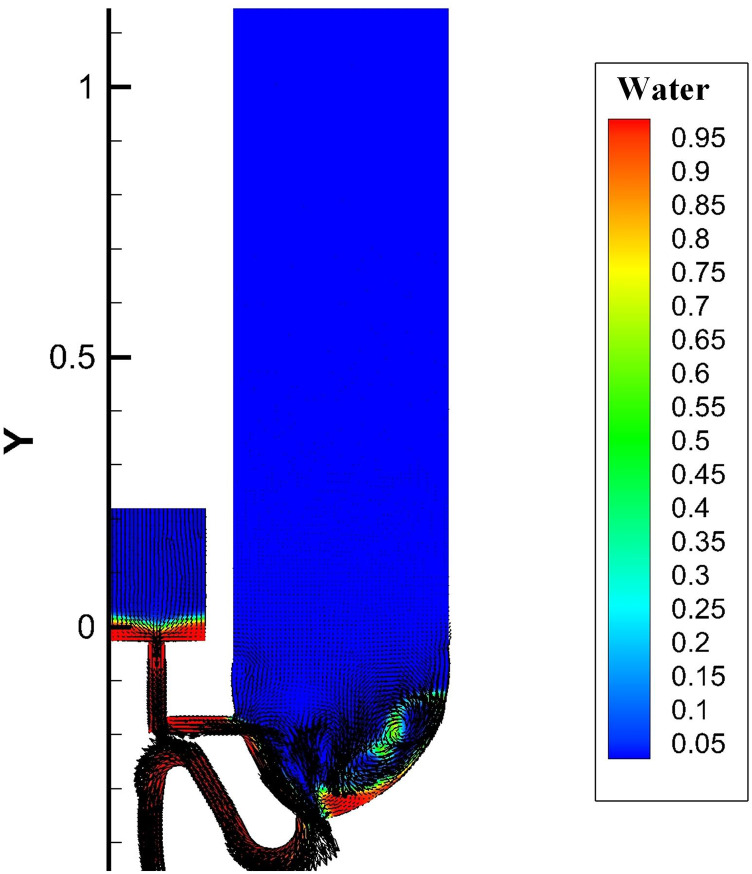
Single-inlet flushing process. Multimedia view: https://doi.org/10.1063/5.0013318.110.1063/5.0013318.1

**FIG. 5.**
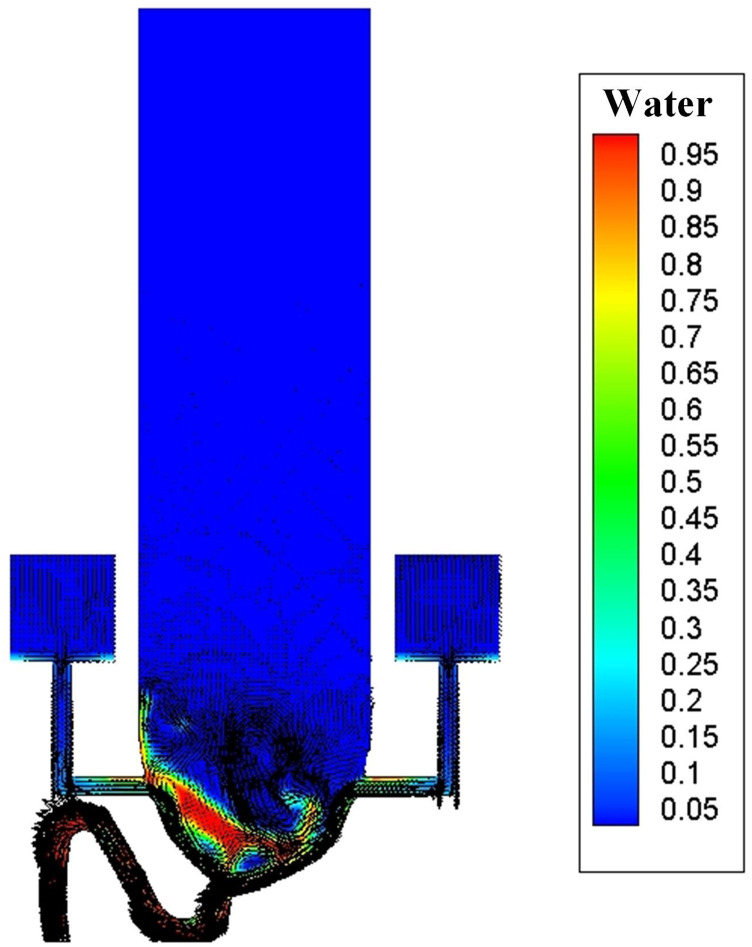
Annular flushing process. Multimedia view: https://doi.org/10.1063/5.0013318.210.1063/5.0013318.2

Dynamic videos of these two flushing processes are shown in Figs. 4 and 5 (multimedia views) (these videos are presented in a mixed form of the water-phase fraction and velocity vector), where approximately two stages of the flushing process can be seen: (1) a drainage stage (before 1.7 s ± 0.1 s) and (2) a late drainage stage (after 1.7 s ± 0.1 s). This classification is based on the fact that, after 1.7 s ± 0.1 s, a large bulk of the liquid is drained out from the bowl area.

**FIG. 6.**
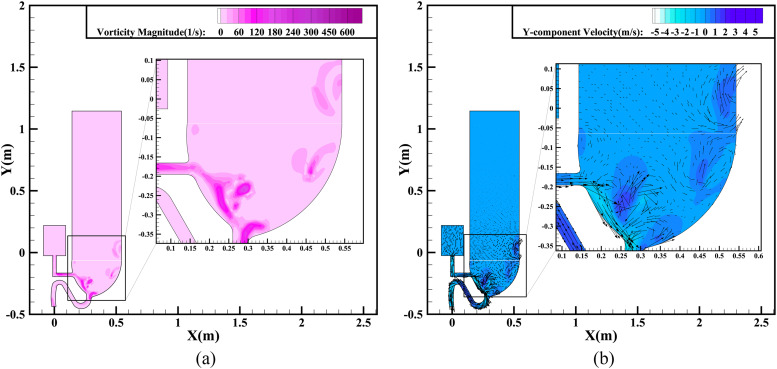
Simulation results of single-inlet flushing at 0.6 s: (a) vorticity magnitude contours; (b) *Y*-component velocity contours and vectors.

**FIG. 7.**
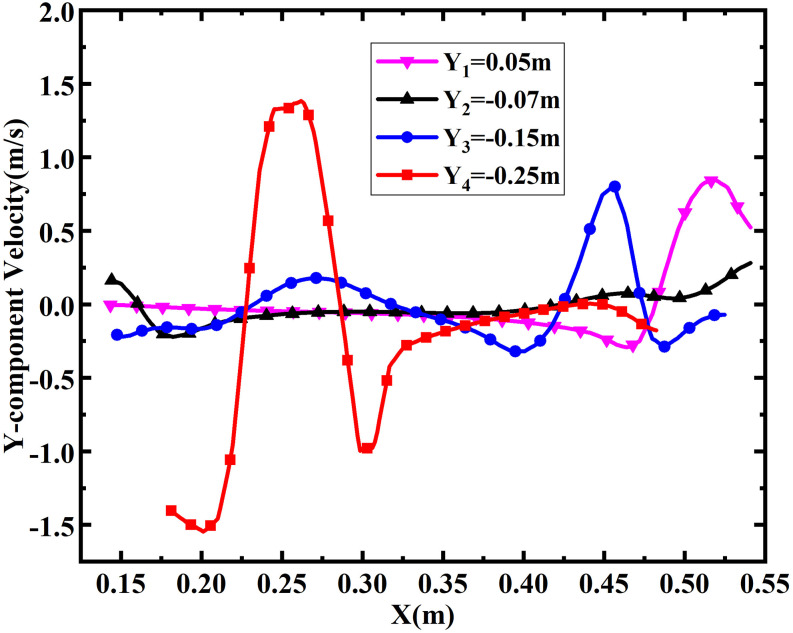
*Y*-component velocity distribution at different locations within the single-port toilet at 0.6 s.

Figure 6 presents contours of the vorticity magnitude and the *Y*-component velocity of the single-inlet flushing at a time 0.6 s after the start of flushing. This represents the beginning of the siphon phenomenon, resulting in the liquid phase flowing out of the sewage pipe, with the pressure and weight of the mixed liquid increasing continuously. As the water pours from the left port, it impacts on the left wall of the bowl, and the splashing liquid washes the right wall, forming vortices near that wall. At the same time, the vortices move continuously upward along the wall surface under the action of the inertia force. Therefore, an airflow vortex also appears in the air zone above the toilet seat, as shown in Fig. 6(a). In addition, there are obvious changes in the magnitude and direction of the velocity in the air zone resulting from the turbulence of the air vortex, as shown in Fig. 6(b). For an easy quantitative analysis, the *Y*-component velocities at different locations within the single-port toilet are displayed in Fig. 7, where the maximum *Y*-component velocity is 1.5 m/s, which occurs at *Y* = −0.25 m, where the vortex intensity is greatest.

**FIG. 8.**
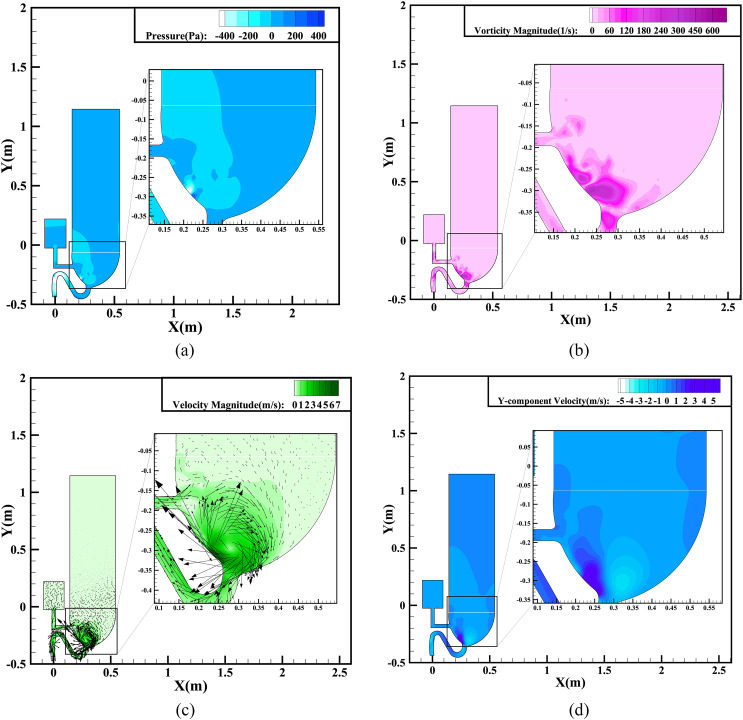
Simulation results for single-inlet flushing at 1.8 s: (a) pressure contours; (b) vorticity magnitude contours; (c) velocity magnitude contours and vectors; (d) *Y*-component velocity contours.

**FIG. 9.**
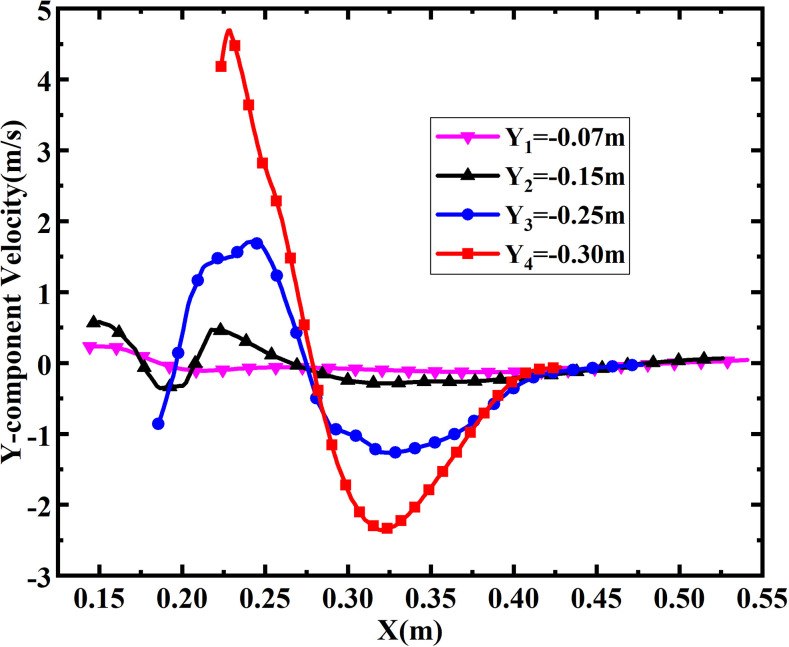
*Y*-component velocity distribution at different locations within the single-port toilet at 1.8 s.

As time goes on, the late stage is entered, when the entire bulk of liquid in the bowl has been drained and the siphon phenomenon ends. Figure 8 illustrates the flow dynamics at a typical time of 1.8 s. Although much of the liquid has been delivered out of the bowl, the liquid–air interaction under the inertia force continues, resulting in the greatest vorticity of 376.12 s^−1^ during flushing, as shown in Fig. 8(b). This generates a strong centripetal force and results in the maximum velocity gradient of the whole region occurring at this moment. The very high vorticity is due mainly to the high speed of the airflow from the port as the liquid runs out of the tank. The pressure contours shown in Fig. 8(a) exhibit similar features to the vorticity distribution according to Bernoulli’s principle, where areas with a higher kinetic energy have a lower pressure energy. Figure 8(c) even shows a huge airflow vortex rotating clockwise within the bowl. The rotational speed is sufficiently large for the vortex center to form a cavity, as can be seen in Fig. 8(c). Figure 8(d) demonstrates that the location with the greatest absolute *Y*-component velocity appears close to the strongest vortex. According to Fig. 9, the maximum *Y*-component velocity appears at *Y* = −0.3 m, which is located on the left side of the cavity, and the maximum value can be up to 4.8 m/s.

**FIG. 10.**
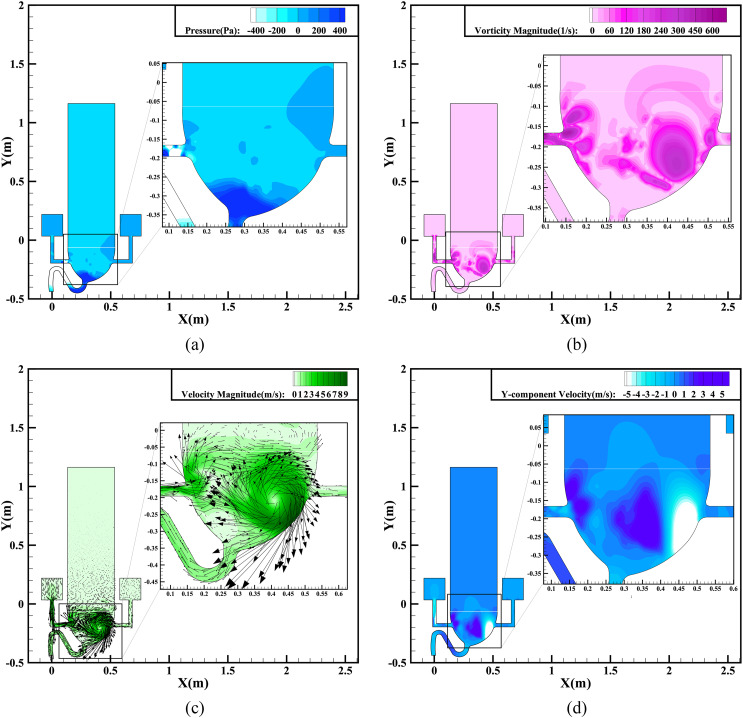
Simulation results for annular flushing at 1.4 s: (a) pressure contours; (b) vorticity magnitude contours; (c) velocity magnitude contours and vectors; (d) *Y*-component velocity contours.

**FIG. 11.**
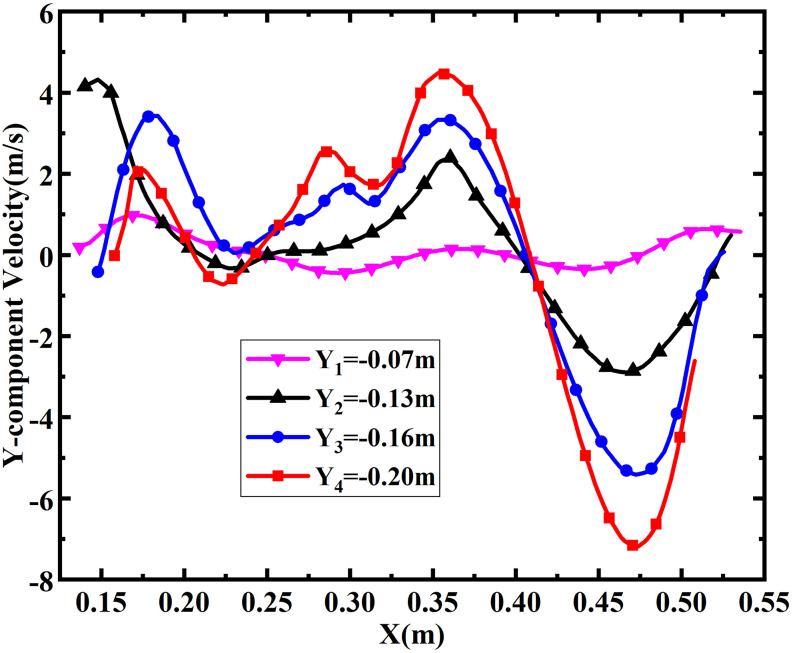
*Y*-component velocity distribution at different locations within the double-port toilet at 1.4 s.

Annular flushing exhibits similar vortex phenomena, as can be seen in Fig. 10, where the data are extracted at a time of 1.4 s, which is classified as being in the first stage. As the water pours from the two symmetrical ports at the same time, it impacts on the water seal, and the mixed liquids alternately scour each side of the wall, forming a large number of vortices near the wall surface, as shown in Fig. 10(b). Among these, the greatest vorticity is up to 484.12 s^−1^, which is a 28.7% enhancement compared with that in single-inlet flushing. This maximum value appears near the port on the right wall. This largest vortex is caused mainly by two high-speed airflows, along with the water coming from the tanks in opposite directions and mixing with the liquid already in the basin. This generates a strong centripetal force and results in the maximum velocity gradient of the whole region occurring at this moment, as shown in Fig. 10(d). From Fig. 10(c), it can be seen that the largest vortex also rotates clockwise, and the velocity is sufficiently high that a cavity forms in the center region. The pressure contours shown in Fig. 10(a) are again similar to the vorticity distribution, which also follows Bernoulli’s principle. Similar to the results for single-inlet flushing at 1.8 s, Fig. 10(d) demonstrates that the location with the greatest absolute *Y*-component velocity appears close to the strongest vortex. According to Fig. 11, the range of fluctuations of the *Y*-component velocity can be as high as 12 m/s, with the maximum magnitude approaching 5 m/s. This occurs at *Y* = 0.2 m, which is located on the left side of the vortex cavity.

**FIG. 12.**
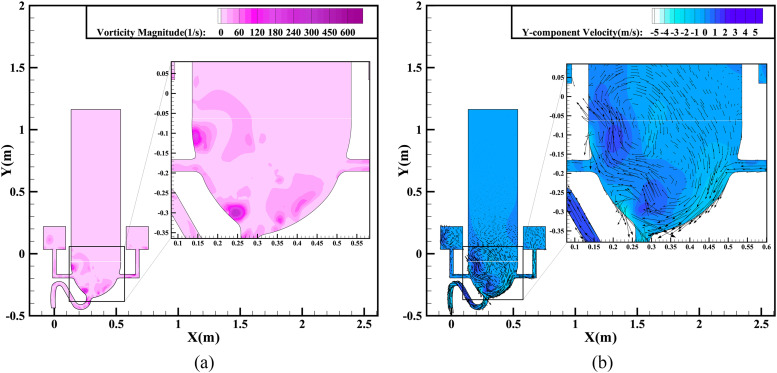
Simulation results for annular flushing at 1.8 s: (a) vorticity magnitude contours; (b) *Y*-component velocity contours and vectors.

With the large bulk of liquid in the bowl drained, the annular flushing enters the late stage, as shown in Fig. 12, where the data are extracted at a time of 1.8 s. As the air continues to interact strongly with the impacting water, an airflow vortex still exists in the bowl, as shown in Fig. 12(a), and continues to generate centripetal force. According to Fig. 12(b), the velocity gradient changes together with the vorticity distribution. The turbulence of the air vortices, shown in Fig. 12(a), results in obvious changes in the magnitude and direction of the velocity in the air zone, even above the toilet seat.

Comparison of the flow dynamics of the two flushing processes reveals both similarities and differences. No matter what kind of flushing is used, airflow vortices will appear in the bowl during flushing, and the centrifugal forces generated by these will give rise to a high airflow speed. The resulting turbulence will disturb the magnitude and direction of the velocity in the air zone above the toilet area. Therefore, it is reasonable to assume that the high-speed airflow will expel aerosol particles from the bowl to regions high in the air above the toilet, allowing viruses to spread indoors causing risks to human health. Compared with single-inlet flushing, annular flushing causes stronger turbulence with a higher *Y*-component velocity as a greater amount of flushing water with a higher impact energy enters the bowl in the first stage. It is also found that the maximum *Y*-component velocity occurs at the center of the bowl in the annular flushing process but at the edge of the bowl in single-inlet flushing. This can be attributed to the fact that, in the annular flushing model, water is supplied from two opposite ports, as a result of which the two streams of water collide in the middle of the bowl to generate a high-speed upward flow.

### B. Particle movement analysis and discussion

**FIG. 13.**
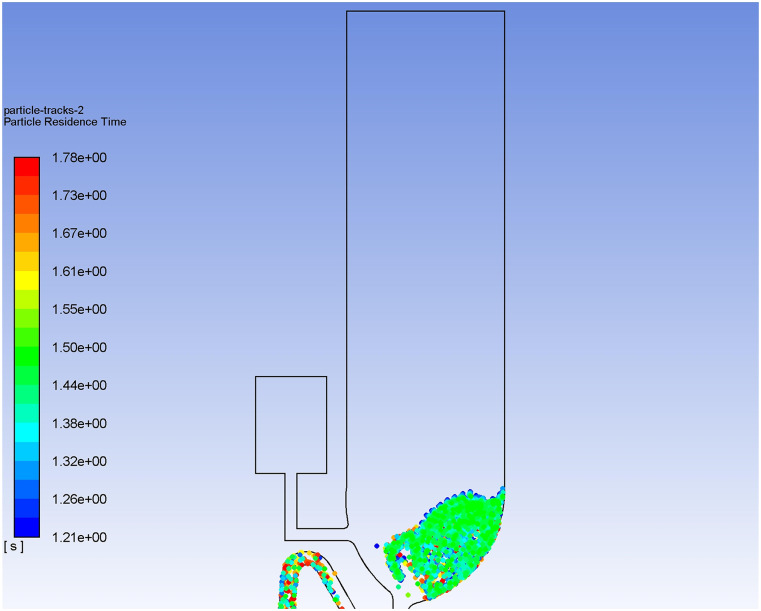
Dynamic virus particle movement during single-inlet flushing. Multimedia view: https://doi.org/10.1063/5.0013318.310.1063/5.0013318.3

**FIG. 14.**
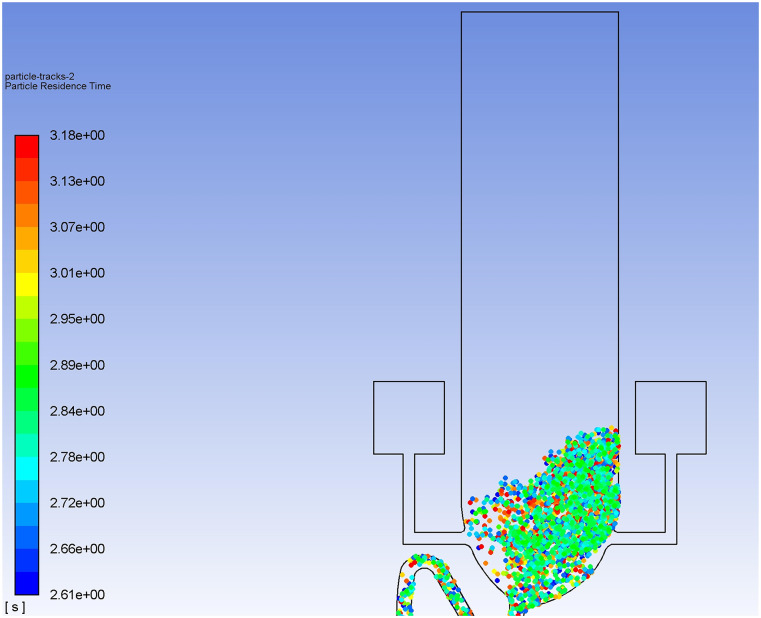
Dynamic virus particle movement during annular flushing. Multimedia view: https://doi.org/10.1063/5.0013318.410.1063/5.0013318.4

**FIG. 15.**
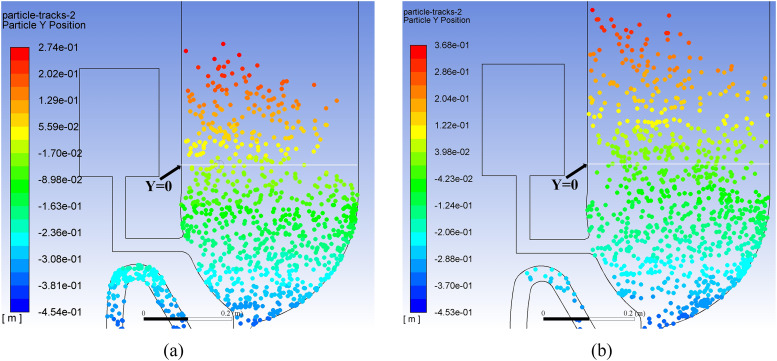
Discrete particle *Y*-position distribution for single-inlet flushing at a time of (a) 35 s and (b) 70 s.

Dynamic virus particle movements during the flushing process of the two toilet models are shown in Figs. 13 and 14 (multimedia views). Without exception, massive upward particle transport is observed for both flushing processes. Figure 15 displays the particle *Y*-position distribution for the single-inlet flushing process in the post-flushing time period to show how far particles can move during one-shot flushing. It can be seen in Fig. 15(a) that the *Y* position of the highest particle can reach 27.4 cm at a time of 35 s and in Fig. 15(b) that it can reach 36.8 cm at 70 s. Because the *Y* position of the ground can be estimated as −45 cm, the actual heights of these particles are 72.4 cm at 35 s and 81.8 cm at 70 s. The upward velocity can be estimated to be 0.27 cm/s in the post-flushing period. According to a statistical calculation, 2700 particles are brought out of the toilet during a one-shot single-inlet flushing at a time of 70 s.

**FIG. 16.**
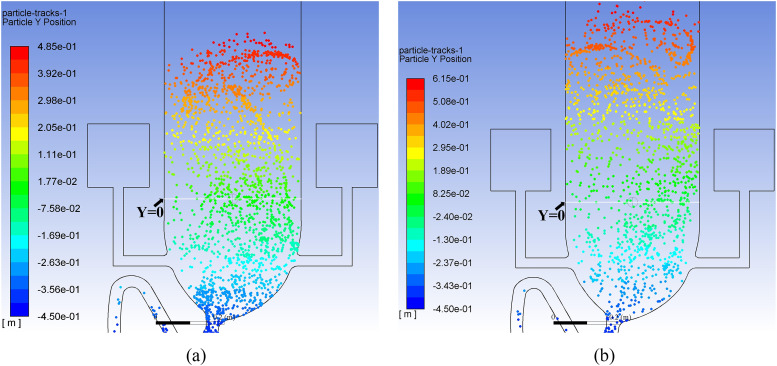
Discrete particle *Y*-position distribution for annular flushing during the post-flushing period at a time of (a) 35 s and (b) 70 s.

Figure 16 illustrates the huge spread of aerosol particles after a long period of time. As can be seen in Fig. 16(a), the *Y* position of the highest diffused particle in the computational domain can reach 48.5 cm at a time of 35 s. The actual height of this particle is 93.5 cm. Furthermore, Fig. 16(b) shows the result at a time of 70 s, when the actual maximum height of a diffused particle is 106.5 cm, which means that the *Y*-component velocity can be 0.37 cm/s, even a long time since the last flushing, which is a 37% enhancement compared with the case of single-inlet flushing. Note that this information is extracted from the particles remaining in the computational zone. The number of escaped particles during annular flushing is calculated to be 1511 (25% of the total number) at a time of 35 s. It can be imagined that the velocity will be even higher when a toilet is used frequently, such as in the case of a family toilet during busy times or a public toilet in a densely populated area. It is statistically estimated that nearly 60% of the total aerosol particles (including those escaping from outlet 1) rise above the toilet seat. This is 33.3% larger than that in the case of single-inlet flushing.

## VI. CONCLUSION

Toilets are a daily necessity but also become dangerous if used improperly, especially against the current scenario of a global pandemic. This paper has used the CFD approach to clarify how toilet flushing can promote virus transmission. The flushing processes of two different types of toilets (single-inlet flushing and annular flushing) have been simulated, and in particular, the fluid flow characteristics and the movement of aerosol particles during flushing have been investigated. Several alarming conclusions can be summarized as follows:

• Strong turbulence has been observed to be generated by both flushing methods.
• An upward velocity of as much as 5 m/s is produced, which is certainly capable of expelling aerosol particles out of the toilet bowl.
• Some 40%–60% of the total number of particles can rise above the toilet seat to cause large-area spread, with the height of these particles reaching 106.5 cm from the ground.
• Even in the post-flushing period (35 s–70 s after the last flushing), the upward velocity of the diffused particles can reach 0.27 cm/s–0.37 cm/s, and they continue to climb.
• The data analysis indicates that given the same amount of water and the same gravitational potential energy, annular flushing causes more virus spread.

Faced with these alarming results, we advocate several safe procedures to adopt when using a toilet:

1. Put the toilet lid down before flushing, which can basically prevent virus transmission.
2. Clean the toilet seat before using it, since floating virus particles could have settled on its surface.
3. Wash hands carefully after flushing, since virus particles may be present on the flush button and door handle.

This paper may also enlighten toilet manufacturers and prompt them to produce better designed toilets in which the lid is automatically put down before flushing and cleaned before and after flushing.

## AUTHORS’ CONTRIBUTIONS

Y. Li and J.-X. Wang contributed equally to this work.

## DATA AVAILABILITY

The data that support the findings of this study are available from the corresponding author upon reasonable request.
